# Spontaneous Isolated Celiac Artery Dissection in a Young Woman

**DOI:** 10.7759/cureus.71539

**Published:** 2024-10-15

**Authors:** Mao Tanabe, Seigo Urushidani

**Affiliations:** 1 Emergency and Critical Care Center, Kurashiki Central Hospital, Kurashiki, JPN

**Keywords:** abdominal pain, celiac artery dissection, computed tomography angiography, dysmenorrhea, sicad, spontaneous isolated celiac artery dissection, women

## Abstract

Spontaneous isolated celiac artery dissection (SICAD) is a rare phenomenon with an unclear pathogenesis. It is more common in men, with a predilection for those in their 50s. A 39-year-old woman with epigastric and back pain visited our emergency department (ED). She had no cardiovascular risk factors. On her first visit to the ED, no signs of peritonitis were noted, and her vital signs were normal. Although a non-contrast abdominal computed tomography (CT) scan was performed, the cause of her abdominal pain remained unidentified. Due to persistent mild-to-severe pain, the patient visited our ED again the following day. Despite repeated blood tests showing no abnormalities, we considered that her abdominal pain was of vascular origin. We performed a contrast-enhanced CT scan, which revealed poor contrast enhancement in the celiac artery without intestinal ischemia. Based on the medical history and CT findings, the patient was diagnosed with SICAD. We consulted a cardiovascular surgeon who advised conservative management. The patient was discharged on the 10th day after admission, and no recurrence of abdominal pain was reported. SICAD is a rare but potentially serious condition that can also occur in younger women. While certain risk factors for SICAD have been identified, some young patients may not have traditional risk factors. Physicians should consider SICAD as a differential diagnosis even in young women when their abdominal pain is persistent without other organ-specific signs.

## Introduction

Spontaneous isolated celiac artery dissection (SICAD) is a rare phenomenon with an unclear pathogenesis. While SICAD is more common in men and typically affects individuals in their 50s, rare cases in young women have been reported, and little is known about such cases [1-3]. Risk factors for celiac artery dissection include high blood pressure, trauma, pregnancy, and atherosclerotic vascular disease [1]. Here, we describe a rare case of SICAD in a young woman with no known risk factors.

## Case presentation

A 39-year-old woman presented to the emergency department (ED) twice due to abdominal pain. She experienced epigastric and back pain a day before her first ED consultation. It began as abdominal bloating and then gradually shifted to epigastric and back pain. The pain was described as a continuous and squeezing sensation. Nausea was present as an accompanying symptom, but there was no diarrhea. Despite taking over-the-counter acetaminophen, the symptoms did not improve. The patient had no cardiovascular risk factors and was not on any regular medications. Although the patient had dysmenorrhea with heavy menstrual bleeding, she did not visit the hospital regularly. The patient and her husband denied pregnancy, and she had finished her regular menstrual cycle the day before the ED visit.

On the first ED visit, no signs of peritonitis were observed during the abdominal examination, and the patient’s vital signs were normal. Electrocardiography showed no ischemic changes. Blood test results revealed no significant abnormalities. Given the patient’s age and risk factors, we determined that the risk of vascular disease was low. Therefore, we performed non-contrast abdominal computed tomography (CT), but could not identify the cause of the abdominal pain. The patient was discharged after symptomatic treatment. However, owing to persistent pain with a Numeric Rating Scale score of 7/10, the patient decided to seek medical attention again the following day.

**Figure 1:**
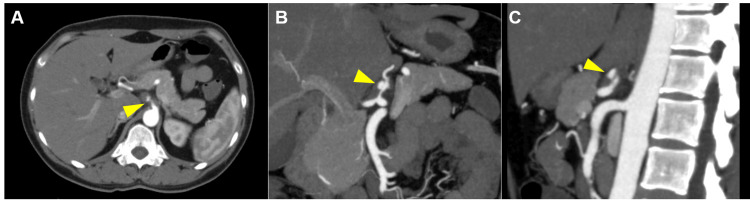
Contrast-enhanced CT of the celiac artery Dissection and narrowing of the celiac artery are seen (yellow arrowheads). A: Axial view, B: coronal view, C: sagittal view.

During the second consultation, the patient’s vital signs were as follows: blood pressure - 128/73 mmHg, respiratory rate - 18 breaths per min, pulse rate - 92 beats per min, SpO2 - 98% on room air, temperature - 36.5°C, and Glasgow Coma Scale - E4V5M6. Although tenderness was observed in the upper abdomen, no signs of peritoneal irritation were noted. We checked the blood tests again. However, no abnormalities were found in the blood tests, including C-reactive protein, lactate and D-dimer. Due to persistent moderate-to-severe abdominal pain without diarrhea or hematuria, we considered vascular disease as the remaining critical differential diagnosis. We performed a contrast-enhanced CT scan (arterial and venous phasing) to investigate for vascular lesions. The scan revealed poor contrast enhancement in the celiac artery without intestinal ischemia (Figure 1). Based on the medical history and CT findings, the patient was diagnosed with SICAD.

We consulted a cardiovascular surgeon and the patient was urgently admitted. Since there were no signs of vascular rupture or intestinal ischemia on the contrast-enhanced CT, conservative management was chosen. Carvedilol was administered to prevent blood pressure elevation, and continuous fentanyl administration was initiated for the persistent abdominal pain. The pain improved on the third day of admission, and the analgesic was given as needed. On the eighth day after admission, a repeat contrast-enhanced CT scan was performed, which revealed that the diameter of the celiac artery and its true lumen showed no significant change compared to the findings at admission. Surgery was deemed unnecessary. The patient was discharged on oral carvedilol to prevent blood pressure elevation on the 10th day. A follow-up contrast-enhanced CT scan performed six months later revealed improvement in the celiac artery dissection and dilation. After one year, no recurrence of symptoms was reported.

## Discussion

We report a case of SICAD that occurred in a young woman of reproductive age. Over 80% of patients with SICAD are males. It is more common in Asians than in Caucasians, with a peak incidence in the fifth decade of life [1,2]. Patients with SICAD often have a history of hypertension and smoking [1]. Although rare, congenital disorders that weaken the arterial wall may increase the risk of SICAD [4]. However, our patient had no history of congenital cardiovascular disorders. The case report and literature review by Tana et al. searched 180 studies and found only three cases of SICAD in women under 50 years of age, including their own. These patients had common vascular risk factors such as hypertension or hyperlipidemia [5]. Like in the current case study, many symptomatic patients complain of abdominal pain [2,5]. Approximately 20-50% of patients with SICAD are asymptomatic and incidentally diagnosed while undergoing imaging examination for other purposes. Our patient was a young woman with no medical history of common cardiovascular risk factors who complained of abdominal pain. The decision to perform contrast-enhanced CT was driven by the need to exclude other critical diseases rather than by specific signs. 

Hormonal contraceptives, menopause, and post-pregnancy conditions can induce estrogen-mediated negative feedback in the hypothalamic-pituitary-gonadal axis, indirectly altering cardiac physiology and leading to changes in coagulation, atherogenic oxidative stress, and vascular tone regulation [6]. Despite having symptoms of dysmenorrhea with heavy menstrual bleeding, the patient had no history of gynecological consultations. Thus, although our patient did not take oral contraceptives at all, hormonal imbalances causing vascular stress may have been present. In addition, Dubey et al. suggested that heavy menstrual bleeding might be associated with cardiovascular disease in women under 40 years of age [7]. Though available literature does not specify dysmenorrhea, hormonal imbalance, or oral contraceptive use as causes for SICAD, based on our case report, we propose that hormonal imbalance or heavy menstrual bleeding could cause this condition in women.

SICAD without signs of visceral malperfusion may be managed conservatively [1,3]. However, intervention with endovascular treatment or open surgery may be required in some patients with thromboembolic complications, aneurysm rupture, persistent or refractory abdominal pain, or unstable vital signs [1,5]. Therefore, precise diagnosis and evaluation of this condition in patients presenting with abdominal pain is important.

## Conclusions

SICAD is a rare but potentially serious condition that can occur in young women. While certain risk factors for SICAD have been identified, some young patients may not have the traditional risk factors. Physicians should consider SICAD as a differential diagnosis even in young women when their abdominal pain is persistent without other organ-specific signs. 
